# Analysis of How the Term “Shall” Is Understood and Applied to the EU-HTA Regulation

**DOI:** 10.3390/jmahp14030042

**Published:** 2026-07-27

**Authors:** Mondher Toumi, Samuel Aballéa, Bruno Fallissard, Claude Dussart, Pascal Auquier, Laurent Boyer

**Affiliations:** 1 CEReSS/UR3279—Health Services Research and Quality of Life Center, Aix-Marseille University, 13385 Marseille, France; pascal.auquier@univ-amu.fr (P.A.); laurent.boyer@univ-amu.fr (L.B.); 2 Inovintell, Mathenesserlaan 447B, 3023 GJ Rotterdam, The Netherlands; sab@inovintell.com; 3 Centre de Recherche en Épidémiologie et Santé des Populations, INSERM U1018, Université Paris-Saclay, 94807 Villejuif, France; bruno.falissard@gmail.com; 4 UR 4129 P2S Parcours Santé Systémique, Université Claude Bernard Lyon 1, 7–11 Rue Guilllaume Paradin, 69008 Lyon, France; claude.dussart@chu-lyon.fr; 5 Pharmacie Centrale, Hospices Civils de Lyon, 57 Rue Francisque Darcieux, 69230 Lyon, France

## 1. Introduction

The European Health Technology Assessment (EU-HTA) Regulation extensively uses the word “shall”, and its use has caused confusion among audiences unfamiliar with legal terminology.

In everyday language, “shall” often does not imply obligation in the way that “must” does: saying “I shall go to the market” does not suggest obligation, whereas “I must go to the market” implies a requirement imposed by oneself or someone else.

In legal and regulatory texts, the term “shall” is traditionally used to denote an obligation or duty. Unlike the permissive “may”, which implies discretion, “shall” generally indicates that an action is mandatory [1]. Black’s Law Dictionary notes that “shall” is generally imperative or mandatory in legal contexts, effectively meaning “must”; however, it also cautions that sometimes “shall” may be construed as permissive or directive, or as simply an indication of the use of the future tense, depending on the context [2]. The interpretation of “shall” can differ depending on jurisdiction and context. Modern drafting practices have increasingly examined the term due to its ambiguities, as experts have identified several different usages of “shall” in legal texts.

This paper aims to clarify the term “shall” from general legal texts, EU legislation, and the EU-HTA Regulation. It examines whether using “shall” makes a provision legally binding and enforceable and considers the consequences of non-compliance. This document clarifies the use of “shall” in legal policy documents, distinguishing it from its everyday usage to avoid confusion. It is not a research paper or a legal opinion, but represents an authorized opinion based on references and the authors’ expertise. The authors are not liable for their good faith opinions and readers cannot hold them responsible for decisions made based on this manuscript. They should seek professional legal advice when necessary.

## 2. Interpretation of “Shall” in Ex-EU Jurisdictions

In U.S. law, “shall” denotes a mandatory duty, while “may” is permissive. The Supreme Court confirmed this in Kingdomware Technologies, Inc. v. United States (2016), clarifying that “shall” imposes a requirement [3].

Courts sometimes interpret “shall” as permissive rather than mandatory if it aligns with legislative intent (the purpose that the lawmakers intended when drafting the law) [1]. Recent U.S. legal drafting thus prefers “must” over “shall” for the sake of clarity, and style guides recommend this change because “shall” has multiple meanings; Bryan Garner, for example, identified at least eight distinct interpretations of “shall” in U.S. legal documents [4].

In the UK, “shall” has traditionally been used in legislation to signify mandatory obligations.

In recent decades, the UK (and other Commonwealth jurisdictions like Canada, Australia, and New Zealand) have moved away from using “shall” in new legislation, viewing it as archaic and potentially confusing. The Office of the Parliamentary Counsel (UK) now has an explicit policy to avoid the legislative “shall”. The official drafting guidance states: “Office policy is to avoid the use of the legislative ‘shall’.” [5].

In international law, “shall” is used in treaties to convey binding obligations. When a country ratifies a treaty, it must adhere to pacta sunt servanda (Article 26 of the Vienna Convention), meaning that treaty terms are binding and must be executed in good faith. Thus, any “shall” clause in a treaty is legally enforceable internationally [6,7].

## 3. “Shall” in EU Law

In the EU’s legal texts, “shall” is understood as imposing a legal obligation—it is effectively equivalent to “must” [8]. EU legislative drafting conventions use “shall” in the English versions of regulations and directives to indicate mandatory provisions. In contrast, many English-speaking jurisdictions have adopted the term “must” for clarity [8]. For example, the EU’s English Style Guide explicitly notes that in EU legislation “shall means the same as must”, and it gives examples like “The following products shall be clearly labelled…” as binding requirements [8]. Likewise, prohibitions are phrased as “shall not” (never “may not”) to avoid any ambiguity; for example, “Food shall not be placed on the market if it is unsafe.” [8]. This stylistic choice shows that the EU treats “shall” as the language of command in its legal acts.

EU drafting manuals, like the Joint Practical Guide, stress clarity and consistent terminology, reserving “shall” for obligations. Though some suggest using “must” instead, the Interinstitutional Style Guide still uses “shall” for normative provisions [4]. In EU regulations, “shall” signifies an imperative, legally binding requirement for its addresses.

## 4. The EU-HTA Regulation

The new EU-HTA Regulation (Regulation (EU) 2021/2282) [9] contains numerous provisions using “shall” to mandate actions by Member States (MS) and EU bodies. “Shall” appears a total of 209 times in the EU-HTA Regulation. For instance, it states that “Member States shall appoint members and representatives” to the coordination group, among other duties [10]. Such phrasing is not merely aspirational—it creates a binding legal duty for MS. In EU law, a regulation is directly applicable in all MS, so a clause stating that “Member States shall do X” means that they are obliged to perform X by the force of EU law. There is no discretion to opt out or delay; failure to comply constitutes a breach of EU law.

EU legal interpretation benefits from multilingual consistency—the obligation conveyed by “shall” in English is mirrored by terms in other EU languages that unequivocally signal duty. For example, in the EU-HTA Regulation in French texts, “les États membres sont tenus de” is used as a translation of “shall”, meaning “Member States are obliged to”. This is consistent with several other languages, such as Spanish, German, and Italian, as observed by the authors using automated translation.

Participation in the EU-HTA Regulation is mandatory and legally binding for MS and Health Technology Developers (HTDs) must submit a high-quality Joint Clinical Assessment (JCA) dossier. Below are excerpts from the EU-HTA Regulation [9] in italics to highlight examples of the duties of MS and HTDs, followed by short explanations.

“This Regulation shall be binding in its entirety and directly applicable in all Member States”. MS must comply with the EU-HTA Regulation and fulfill their duties under the law. Non-compliance is a violation of EU law.

“Member States must designate their members to the Coordination Group and inform the Commission of any changes”. Participation is mandatory, not voluntary.

“The members of the Coordination Group shall designate their national or regional authorities and bodies as members of subgroups of the Coordination Group.” Upon their appointment, the members of the Corporate Governance (CG) committee have several legal obligations as outlined by statutory regulations.

“The health technology developer shall submit the dossier to the Commission in accordance with the submission request made pursuant to paragraph 1”. It is a legal and enforceable requirement to submit a JCA dossier by HTDs.

“The dossier shall meet the following requirements:

- the submitted evidence is complete with regard to the available studies and data that could inform the assessment;
- the data has been analyzed using appropriate methods to answer all research questions of the assessment;
- the presentation of the data is well structured and transparent, thereby allowing for an appropriate assessment within the limited timeframes available;
- it includes the underlying documentation in respect of the submitted information, thereby allowing the assessor and co-assessor to verify the accuracy of that information.
- The dossier for medicinal products shall include the information set out in Annex I.”

HTDs must submit a dossier and meet all of the legal requirements. They need to show the JCA subgroup that they have complied with these five stipulations.

“Where the Commission finds that the dossier fails to meet the requirements laid down in Article 9(2), (3) and (4), it shall request the missing information, data, analyses and other evidence from the health technology developer (second request). In such a case, the health technology developer shall submit the requested information, data, analyses and other evidence in accordance with the timeframe established pursuant to Article 15”. An HTD is required to address missing information analyses or any other evidence requested by the commission.

“The health technology developer shall not provide any comments on the results of the draft assessment”. HTDs are not involved in the scoping nor in the JCA review.

## 5. Implications of Non-Compliance

If an MS does not follow an obligation stated with “shall” in an EU regulation, it violates EU law. The European Commission can enforce these obligations and start an infringement procedure against the non-compliant MS [11]. This process may lead to referral to the Court of Justice of the EU, which can impose financial penalties on a state that does not comply [11]. Regulations are directly applicable, so the affected parties can invoke them in national courts if the provisions are clear and unconditional. This leaves room to escalate at EU-relevant court. Essentially, “shall” is binding in EU law, with non-compliance leading to legal consequences.

While the law does not specify legal consequences for failing to submit a JCA dossier, the EU Commission can refer the matter to the EU Court of Justice, which may result in legal consequences for an HTD, including fines.

## 6. “Should” in the EU-HTA Regulation

In the EU context, including in the EU-HTA Regulation, “shall” imposes obligations, while “should” appears in recitals or non-binding guidances, indicating political or ethical preferences rather than legal duties. “Should” suggests best practices or advisable actions, but is neither legally required nor binding. The term “should” is used frequently in the EU-HTA Regulation, appearing a total of 87 times.

## 7. “May” in the EU-HTA Regulation

In the context of the EU, including in the EU-HTA Regulation, “shall” is used to impose obligations, whereas “may” typically indicates discretion or permission. It characterizes an optional or permissive action. “May” grants the power or authority to do something but does not require it. This term appears infrequently (19 times) in the EU-HTA Regulation.

## 8. EU Law Corpus

A law in the EU context is a broader term that refers to all binding legal acts adopted by EU institutions, including regulations, directives, and decisions. EU law is made up of primary law (treaties) and secondary law (such as regulations, directives, and decisions). While regulations are immediately applicable once in force, other forms of EU law, like directives, require MS to achieve certain results but allow them to choose the means of implementation [12]. They become part of national law and can be enforced through the national courts of each MS from the time that they come into force.

A regulation in the EU is a binding legislative act that is directly applicable and enforceable in all MS as soon as it comes into force. It does not require any national legislation to be implemented and applies in its entirety across the EU. Regulations override any conflicting national laws and ensure uniformity in their subject matter throughout the Union [12]. Regulations become part of national law and can be enforced through the national courts of each MS from the time that they come into force [12].

The EU-HTA Regulation is therefore in force from the 12 January 2025; it is a binding legislative act, is directly applicable in all EU MS automatically and uniformly, and it overrides national laws.

## 9. Conclusions

The term “shall” may sometimes carry some ambiguity in legal contexts, but it is generally equivalent to “must”. It defines obligations and should not be interpreted as suggesting that an action is optional or discretionary. Due to the potential multiple meanings of “shall” identified in the legal and regulatory corpus, lawmakers have increasingly substituted “shall” with “must”.

However, within the EU’s legal texts, “shall” remains the reference and is understood to impose a legal obligation that is effectively equivalent to “must”. There is no ambiguity regarding this interpretation. Consequently, participation in all EU-HTA requirements is mandatory and legally binding for MS and HTDs. Non-compliance exposes MS and HTDs to legal enforcement and penalties.

The EU-HTA Regulation displays all attributes of regulation and is enforceable immediately upon entry into force. Thus, the EU-HTA regulation is imposed on MS and HTDs from the 12 January 2025.

There is to be no doubt regarding the enforcement of the EU-HTA Regulation on MS and the legal liability for not applying the regulation.

## Data Availability

No new data were created or analyzed in this study. Data sharing is not applicable to this article.

## Abbreviations

The following abbreviations are used in this manuscript:

CG	Corporate Governance
EU	European Union
EU-HTA	European Health Technology Assessment
HTDs	Health Technology Developers
JCA	Joint Clinical Assessment
MS	Member States
